# Methodology of “14–7” Program: A Longitudinal Follow-Up Study of the Pediatric Population and Their Families Exposed to the Terrorist Attack of Nice on July 14^th^, 2016

**DOI:** 10.3389/fpsyt.2019.00629

**Published:** 2019-09-12

**Authors:** Morgane Gindt, Susanne Thümmler, Andréa Soubelet, Fabian Guenolé, Michèle Battista, Florence Askenazy

**Affiliations:** ^1^Hôpitaux Pédiatriques de Nice CHU-Lenval, Nice, France; ^2^Université Côte d’Azur, Nice, France; ^3^Centre Hospitalier Universitaire de Caen, Caen, France

**Keywords:** post-traumatic stress disorder, children, longitudinal studies, cohort, psychiatry

## Abstract

**Introduction:** After a traumatic event, children and adolescents may present several clinical consequences, the most common being Post-Traumatic Stress Disorder (PTSD). Most children and adolescents with PTSD have comorbid disorders, such Attention Deficit Hyperactivity Disorder, depression, attachment and anxiety disorders, sleep disturbances and behavior problems. However, epidemiological studies on the development of PTSD and other psychiatric disorders in children and adolescents as a consequence of a terrorist attack and mass murder are lacking. Long-term follow-up of exposed children and adolescents will help identify risk and protective factors of developing psychiatric and psychological conditions after exposure to traumatic events or situations. The main objective of this article is to present the methodology of “14–7” program. The aim of “14–7” program is to characterize the risk and protective psychosocial factors affecting the clinical evolution of a pediatric population sample, exposed to the terrorist attack of July 14^th^, 2016 in Nice.

**Method and Analysis:** “14–7” program is a multicentre longitudinal cohort study. Major inclusion criteria are children and adolescents exposed to the terrorist attack and aged under 18 years on July 14^th^, 2016. These children and adolescents will be compared to a non-exposed to the “14–7” terrorist attack group, matched on age and gender. Participants will be assessed at baseline (T1), 2 years (T2) and 5 years (T3) after the initial assessment (T1), and every 5 years until they are 25 years old. Multiple domains are assessed: 1) mental health disorders, 2) intensity of PTSD symptoms, 3) intensity of comorbid symptoms, 4) quality of the parent–child relationship, 5) intelligence quotient, 6) parental symptoms. We will also establish a biological collection of saliva samples, magnetic resonance imaging (MRI) and actigraphy data collection. Main analyses comprise analyses of variance and regression analyses of predictors of clinical evolution over time.

**Ethics and Dissemination:** The National Ethics Committee “NORD OUEST III” approved the “14–7” Program protocol (number 2017-A02212-51). All patients and their caregivers signed informed consent on enrolment in the “14–7” Program. Inclusions started on November 21^st^, 2017. Three hundred thirty-five individuals have been included (191 children and adolescents, 144 parents).

**Clinical Trial Registration:** www.ClinicalTrials.gov, identifier NCT03356028

## Background

On 14 July 2016 in Nice, celebration day in France, a terrorist attack happened. Many children and their families were impacted physically and psychologically. At least 30,000 people were present on the Promenade des Anglais (the attack site). This is the most important terrorist attack against families and children in France.

Eighty-six people died in the attack, including 10 children, the youngest being 4 years old. At least 55 children and adolescents were bereaved because of this crime. From 14^th^ 2016 to the beginning of the “14–7” Program (November 21^st^, 2017), 827 outpatients children, aged less than 18 years old, have consulted the child and adolescent psychiatry service as results of clinical consequences of this attack (1).

After being exposed to a traumatic event, children and adolescents may suffer from several clinical conditions (2). Among them, the most common is post-traumatic stress disorder (PTSD) (2). PTSD includes four main clinical symptoms: flashbacks of the event, avoidance behaviors, alteration of cognition and mood, and neurovegetative overactivation (3). Moreover, children and adolescents have specific clinical symptoms, such as general fears, developmental regression or traumatic re-enactment through play (4).

PTSD contributes to the development of many other mental disorders (5, 6). Around 75% of children and adolescents with PTSD have comorbid disorders. The main comorbidities identified in the pediatric populations are anxiety disorders, attention-deficit-hyperactivity disorder and depression (7, 8, 9). In addition, children and adolescents might present reactive attachment disorder, sleep disturbances and behavior problems (such as oppositional defiant disorder) (8).

Rates of PTSD in children and adolescents exposed to a potentially traumatic event have been shown to vary between 6% and 20% depending on several factors (10). That is, trauma history and exposure, sociodemographic, psychiatric, psychologic, environmental, and biological factors may modulate the development of PTSD.

Concerning trauma history and exposition, it has been reported that individuals who experience multiple trauma are at higher risk of developing chronic PTSD (11). In addition, the degree of violence, the individual experiences during the exposition, is known to influence the development of PTSD (12). However, whether the exposure is direct or indirect does not seem to have an impact on PTSD development, but it may have an effect on its chronicity (13). Repeated trauma cause more PTSD in children and adolescents than single events [35% (14) vs. 18% (15)]. The prevalence of PTSD after a human attack (more than 50% (16, 17) might be much higher than after other traumas [23% to 30%, after a natural disaster (18, 19), 17% after road accidents (15, 20)]. A higher prevalence of PTSD after a mass attack compared to other types of trauma is also described in adults [39% vs 10% (21, 22)].

Among sociodemographic factors, age, sex, socio-economic status (15) and psychosocial resources (23) have been associated with different varying risks of PTSD. Some studies assumed that being a woman or a girl has been found associated with higher risk of PTSD development after a traumatic event (24). Younger children might have a higher prevalence of PTSD compared to older ones [39% for under 6 years versus 33% for 6–11 or 27% 12 years and older (9)]. People with lower socioeconomic status (15) and with depleted psychosocial resources (23) seem at greater risk of PTSD development. Consistent effects were found for these variables in PTSD severity (9.2% more) and PTSD onset (8.75% more), while the results are less obvious concerning racial/ethnic differences in PTSD persistence (5.40% more).

Among psychiatric factors, having anterior mental disorders, and in particular depression, anxious disorders, or PTSD (25) is associated with higher intensity of acute stress reaction and higher risk of peri-traumatic dissociation (26). Moreover, the risk of PTSD chronicization is increased when the child develops PTSD comorbidities (27).

Concerning psychological factors that have been found related to PTSD, the feeling of fear, during trauma and in the first month after the exposition to the traumatic event, is a risk factor implicated in the PTSD development (28). In a similar way, guilt and shame after the event predict the development of this disorder (29). Finally, cognitive alterations, that is, alterations of working memory and executive functioning and attentional biases are correlated with the occurrence and the risk of transition to PTSD chronic state symptoms (30).

Among environmental factors, family reactions during and after the trauma modulate the risk of PTSD development (31). Parental distress as well as parental PTSD and other parental mental disorders conduct to a higher risk of PTSD in children and adolescents (32, 33). On the contrary, quality of familial and social support, available psychological health care, recognition of traumatic impact by the society (31) have been identified as protective factors of PTSD development.

Finally, researches on adults have shown three biological markers of PTSD development: low cortisol 24 h after trauma (34), reduction of the volume of the hippocampus (35) and prefrontal dysfunction (36). Studies underlined that individuals with decreased hippocampus volume and prefrontal dysfunction following exposure displayed more PTSD-related symptoms 1 month after. In contrast, initial smaller hippocampus volume pre-exposure did not have an effect on PTSD-related symptoms (35).

Although several risk factors of PTSD in both children and adults have been documented, most research on risk factors of PTSD development in children and adolescents has focused on a small number of factors. Therefore, there is to date no integrative model of the PTSD development, and of risk of chronicity or relapse. Research on adult PTSD attempts to demonstrate the existence of different PTSD patterns consistent with symptoms, trauma and comorbidity (37) and to understand the temporal courses of PTSD (38). These results have not yet been replicated in children and adolescents.

Because both children and parents were exposed to the event and because of the nature of traumatic event, the prevalence of PTSD in the pediatric population implicated in the Nice attack may be very important.

The primary objective of the “14–7” Program is to characterize the risk and/or protective psychosocial factors affecting the clinical evolution of a pediatric population sample exposed to the terrorist attack of July 14^th^, 2016 in Nice.

Secondary objectives of the “14–7” Program are to examine efficacy of clinical treatments as a function of the developmental age of the child, and to develop recommendations for treatments and therapies.

## Methods/Design

### Ethical Consideration, Funding, and Registration

The “14–7” Program protocol was approved by the National Ethics Committee “NORD OUEST III” (number 2017-A02212-51).

All patients and their legal caregivers signed informed written consent upon enrolment in the study. The “14–7” Program has also been registered with ClinicalTrials.gov (number NCT03356028). Inclusions started on November 21^st^, 2017.

### Objectives

The main objective of “14–7” Program is to characterize the risk an*d/o*r protective psychosocial factors affecting the clinical evolution of a pediatric population sample, after the “14–7” terrorist attack.

Secondary objectives are: 1) assessment of the prevalence and incidence of mental health disorders, 2) assessment of the intensity of PTSD symptoms, 3) assessment of the intensity of comorbid symptoms, 4) assessment of the quality of the parent–child relationship, 5) assessment of the intelligence quotient, 6) assessment of parental symptoms 7) establishment of a biological collection of saliva samples, and 8) establishment of a magnetic resonance imaging (MRI) and actigraphy data collection.

### Study Design

**Figure 1:**
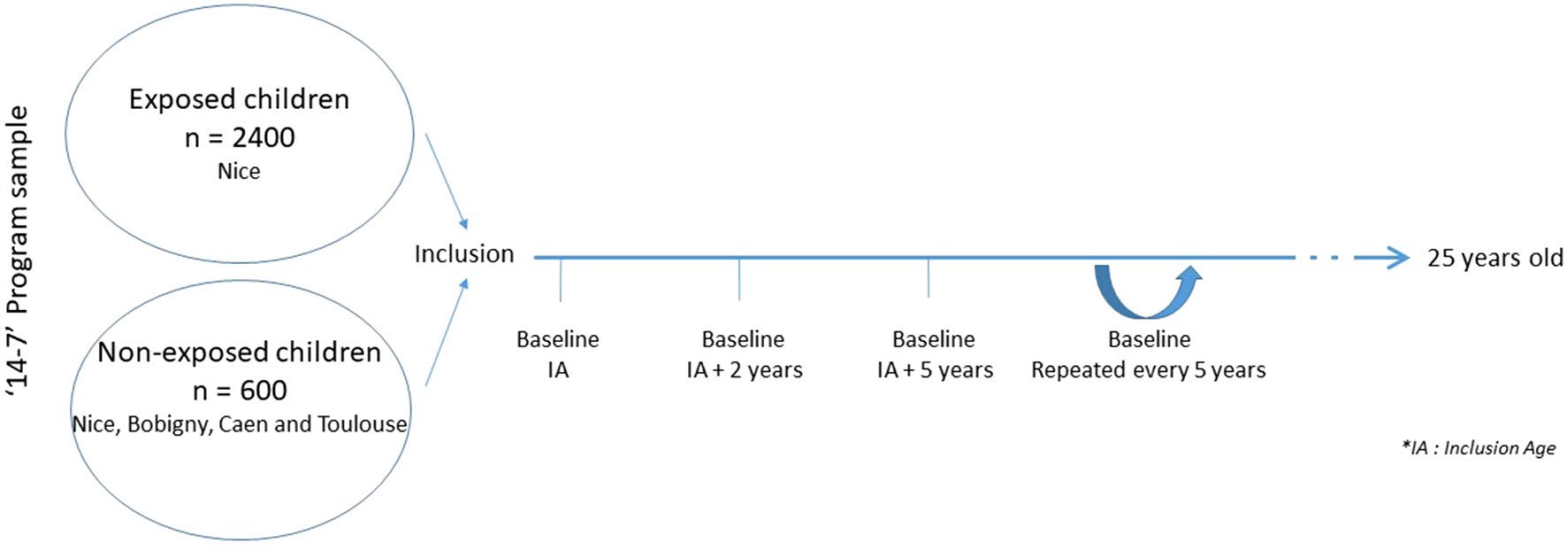
Study design and participants of “14–7” Program.

“14–7” is a longitudinal research involving human beings (i.e., French Law: category 2), with minimal risk and constraints, not involving health products. This search is a longitudinal follow-up study until participants reach the age of 25 years old. Participants will be assessed at baseline (T1), 2 years (T2), and 5 years (T3) after the initial assessment (T1), and every 5 years until they are 25 years old (**Figure 1**). During T1, T2, and T3 salivary samples will be taken from the included children and their parents, for all participants. At T1 and T2, actigraphy and MRI will be proposed for all participants.

Each measurement occasion includes four visits. Measures administered at visits 1, 2, 3, and 4 are summarized in **Table 1**. Assessment will be performed by investigators [child and adolescent psychiatrists, (neuro)psychologists] specifically trained in psychotrauma and child psychiatry.

**Table 1:** “14-7” Program procedure and evaluation criteria for the first study times.

		Selection	V1*	V2*	V3*
**Inclusion criteria**		X			
**Informed consent**		X			
**Psychosocial risks**			X		
**CGI/ CGA** (39, 4)			X		
**Salivary specimen**			X		
**DIPA** (40)	**Psychiatric evaluatio**n		X		
**MiniKid** (41)					
**K-SADS-PL** (42)					
**YCPC** (43)	**PTSD scale**			X	
**CPC** (44)					
**PCL** (45)					
**CDRS** (46)	**Depression scale**			X	
**HDRS** (47)					
**PAS** (48)	**Anxiety scale**			X	
**STAIC** (49)					
**STAI** (49)					
**BITSEA** (50)				X	
**PDEQ** (51)				X	
**Conners** (52)				X	
**IPPA** (53)				X	
**Kidscreen** (54)				X	
**PSI-SF** (55)				X	
**PIPS** (56)				X	
**BECS** (57)	**Cognitive and emotional evaluation**				X
**WPPSI** (58)					
**WISC** (59)					
**WAIS** (60)					

*V1, Visit 1; V2, Visit 2 and V3, Visit 3.

## Participants

Two groups were defined:

- Group 1: children exposed to the “14–7” terrorist attack;
- Group 2: children non-exposed to the “14–7” terrorist attack.

Patient selection and recruitment:

Exposed children are recruited by consultation registry of the Nice CHU Lenval, ORSAN database (a national list of victims from terrorist attack). Controls will be matched on age and sex. Moreover, participants will be recruited *via* press advertisements and National Education. For the control group, categorical screening criteria were used to estimate the number of subjects necessary to reach a statistical power of 80% with a risk of type-I error (α) of 5% for the main outcome variable. Assuming a frequency of development of PTSD of 20% in the general population, to demonstrate a 40% increase in incidence (incidence rate found during violent and intentional traumatic events), it is necessary to include 1,122 participants; that is, 561 child controls. We will then include 600 children (150 per centre) matched on age and sex to the group of exposed children.

### Inclusion Criteria

Exposed group:

- Children, adolescents, and young adults of the Nice region exposed by the terrorist attack and aged under 18 years on July 14^th^, 2016, according to DSM 5.
- Children of pregnant women at the time of the Nice attack.
- Affiliated to a social security scheme;
- Fluent in French;
- Informed written consent of legal guardians and child/adolescent.

Non-exposed group:

- Children, adolescents and young adults, matched in age and gender to exposed group;
- Non-exposed to the “14–7” terrorist attack;
- Affiliated to a social security scheme
- Fluent in French;
- Informed written consent of legal guardians and child/adolescent.

### Non-Inclusion and Exclusion Criteria

Non-inclusion criteria for the Nice cohort and control group were children, adolescents and/or young adults with average intellectual disability (IQ below 50), persons deprived of freedom after judicial or administrative decision, and persons subject to an exclusion period due to participation in other research.

Concerning the exclusion criteria, a request made by the child/adolescent/young adult or his/her parent for no longer participating to the “14–7” Program.

### Measures

Quantitative and qualitative measures are administered to the participants (summarized in **Table 1**):

- Questionnaires regarding psychosocial risks (socio-demographic information; exposure to the traumatic event; care immediately after exposure; current care; social support, media exposure; legal procedure and assistance),
- Semi-structured interviews to assess the main diagnosis in children: MiniKid, Diagnostic Infant and Preschool Assessment (DIPA) or Kiddie—Schedule for Affective Disorders and Schizophrenia (K SADS),
- Salivary specimens for the construction of a biological DNA and RNA database,
- Clinical scales to assess PTSD: Young Child PTSD Checklist (YCPC), Child PTSD Checklist (CPC), or PTSD checklist (PCL),
- Clinical scales to assess anxiety: Preschool Anxiety Scale (PAS), Situational-Trait Anxiety Inventory for children (STAIC), or Situational-Trait Anxiety Inventory (STAI),
- Clinical scales to assess depression: Children’s Depression Rating Scale (CDRS) or Hamilton Depression Rating Scale (HDRS),
- Clinical scales to assess ADH/D (Conners),
- Clinical scales to assess dissociative experiences: Peri-traumatic Dissociation Experiences Questionnaire (PDEQ),
- Evaluation of social aspects: Brief Infant-Toddler Social and Emotional Assessment (BITSEA), Inventory of Parenting and Peer Attachment (IPPA), Kidscreen, parental stress index—short form (PSI-SF), or Post-Trauma Inventory of Parental Style (PIPS),
- Cognitive and socio-affective development for the youngest patients (Cognitive and Socio-Emotional Evaluation Battery: BECS) and intelligence quotient: Wechsler Preschool and Primary Scale of Intelligence (WPPSI), Wechsler Intelligence Scale for Children (WISC), or Wechsler Adult Intelligence Scale (WAIS).
- Parents’ clinical symptoms are also evaluated with the Clinical Global Impression (CGI), Clinical Global Assessment Scale (CGA), PCL, HDRS, STAI, PDEQ, PSI-SF and PIPS.

Clinical scales are all based on DSM-5 classification. They are scientifically validated assessment tools and are often used in clinical settings.

### Data Collection Process

Each investigator at the different study centers is responsible for collecting and entering data on the electronic CRF (Case Report Form) at the time of the visit. The Child and Adolescent Psychiatry University Unit (SUPEA) and the Research Center (Epidemiology and Population Health (INSERM U1018)) will perform data analysis of the database.

### Statistical Analysis

Statistical analyses will be run with R, SPSS, and AMOS softwares.

Descriptive analyses of all the parameters collected at baseline and during follow-up will be conducted.

Correlations will be carried out to examine associations between trauma history and exposition, sociodemographic, psychiatric, environmental, psychological and biological data.

Analyses of variance will be conducted to test the differences between the exposed group and the control group on trauma history and exposition, sociodemographic, psychiatric, environmental, psychological and biological data.

Multivariate models (linear and non-linear regression models, mediation and moderated mediation models) will be carried out to:

1. examine associations between clinical conditions (trauma history and exposition, sociodemographic, psychiatric, environmental, psychological, and biological data), at baseline in children and parents,
2. predict clinical evolution over time with trauma history and exposition, sociodemographic, psychiatric, environmental, psychological and biological variables. Significant predictors from bivariate analysis (at p < 0.05) will then be included in a multivariate regression model. Owing to overlap of assessments, multicollinearity of predictors will be tested and addressed in multivariable models.

Each individual hypothesis will be tested at a significance level of 0.05. However, in order to compensate for the likelihood of incorrectly rejecting a null hypothesis when testing multiple hypotheses (i.e., making a Type I error), methods such as the Bonferroni correction will be used to reduce the likelihood of Type I error when testing multiple comparisons.

These statistics will be conducted on data from the whole cohort and from single groups (i.e., group of exposed children and non-exposed group) and on data from all collection waves.

### Outcomes

The main objective of “14–7” Program is to characterize the risk and/or protective psychosocial factors affecting the clinical evolution to the age of 25 years old of a pediatric population sample exposed to the mass trauma of 14 July 2016 in Nice.

Knowledge concerning risk and/or protective factors will help practitioners to detect children and adolescents at high risk of mental disorders in order to provide efficient follow-up and therapy.

Secondary objectives of the “14–7” Program are to examine efficacy of clinical treatments as a function of the developmental age of the child, and to develop recommendations for treatments and therapies. Results will therefore help future clinicians make evidence-based decisions on which treatment to use, depending on the developmental age of the child, and then help reduce the costs of trauma consequences.

Finally, in France, “14–7” Program should rapidly provide clinicians with adapted and standardized assessment tools validated in the pediatric population of all ages in the French language (e.g., DIPA, YCPC, CPC).

## Discussion

It has been reported that psychiatric disorders such as PTSD have a high prevalence in war-exposed children (61), and more generally in intentional trauma (62). However, research on psychotrauma in terrorism context on pediatric population is rare and/or very fragmented, and there are few reliable data in children and adolescents exposed to traumatic events (63).

Most epidemiological studies have involved either small samples of number of participants or very heterogeneous samples. For example, in some studies, participants have experienced different types of trauma and in other studies, a very small number of factors related to PTSD have been investigated [for a meta-analysis, see Ref. (31)]. In others, very few factors related to PTSD have been investigated such as quality of life [Ref. (31); see also Ref. (18)]. Then, one of the main advantages of the “14–7” Program is that it includes multiple assessments (psychiatric, psychological, psychosocial, biological, and cognitive), on a population of children having experienced the same traumatogenic event. The interest of “14–7” Program is to understand better clinical and cognitive specificities of trauma in pediatric population. Moreover, “14–7” Program integrates a developmental and biological approach.

Children and adolescents are also vulnerable for conduct, attention, and others psychiatric disorders after a PTSD development (8). An acute understanding of the clinical, cognitive, social, and biological consequences of trauma are necessary to orient specific follow up and treatments of the impacted pediatric population.

The main strength of the “14–7” Program is the standardized evaluation and follow-up of a large number of children and adolescents impacted by a terrorist attack in a country in peace, with assessments until the young adult age (25 years).

The American Academy of Child and Adolescent Psychiatry (AACAP) recommends early interventions for children and adolescents to reduce clinical manifestations of trauma (64). AACAP also suggests that relying on the support of the young patient’s family, school, and friends is important, and that psychotherapies should allow the child to talk about the traumatic event in a safe space by speaking, drawing, playing, or writing. Unfortunately, no recommendation on the types of therapies to use and on their duration has been provided so far (64).

### Limitations of the Study

The main limitation of the “14–7” program might be the risk of non-representability of the entire pediatric population impacted by the terrorist attack.

First, individuals exposed to this attack and leaving outside the Nice region will be very difficult to be included. Second, children’ and/or parents’ avoidance behaviour might limit participation to the study. Third, the time lapse between the attack and the start of “14–7” Program does not permit to prospectively analyze clinical manifestations and PTSD during the first year. Nevertheless, questionnaires and diagnostic assessments include retrospective and lifetime evaluations. Finally, participant drop-out occurs in all prospective and longitudinal studies. Attrition not only causes loss of power because of diminishing numbers of participants, but if systematic, this may lead to selection biases and erroneous conclusions being drawn from a study. To prevent attrition, call phoning and newsletters are regularly realized for all participants.

## Ethics Statement

The studies involving human participants were reviewed and approved by National Ethics Committee “NORD OUEST III.” Written informed consent to participate in this study was provided by the participants’ legal guardian/next of kin.

## Author Contributions

MG wrote the manuscript. ST, AS, FG, and MB are the proofreaders. FA is the writer and the coordinator.

## Funding

This work has been supported by the Université Côte d’Azur.

## Conflict of Interest Statement

The authors declare that the research was conducted in the absence of any commercial or financial relationships that could be construed as a potential conflict of interest.
